# Serum Uric Acid Is Associated with CT-Derived Aortic Valve Calcification in Low-Flow, Low-Gradient Aortic Stenosis with Reduced Ejection Fraction

**DOI:** 10.3390/jcdd13070290

**Published:** 2026-06-23

**Authors:** Anıl Avcı, Emre Kipritçi, İbrahim Veyisoğlu, Selahattin Akyol, Emrah Bayam, Serdar Fidan, Ramazan Kargın

**Affiliations:** Department of Cardiology, Koşuyolu Cardiovascular Training and Research Hospital, Istanbul 34865, Türkiye; kipritciemre@gmail.com (E.K.); ibrahimveyisoglu@gmail.com (İ.V.); slhddnakyol@gmail.com (S.A.); emrah_bayam@hotmail.com (E.B.); mdserdarfidan@gmail.com (S.F.); ramazankargin@yahoo.com (R.K.)

**Keywords:** aortic stenosis, heart failure, uric acid, oxidative stress

## Abstract

Background: Low-flow, low-gradient aortic stenosis with reduced left ventricular ejection fraction is a heterogeneous condition with challenging severity assessment. Aortic valve calcification reflects fibro-calcific remodeling, while oxidative stress plays a key role in its pathogenesis. Serum uric acid, a marker of oxidative stress, may be associated with valvular calcification. This study investigated the relationship between serum uric acid levels and aortic valve calcification in this population. Methods: This retrospective study included 85 patients. Aortic valve calcification was quantified using computed tomography with the Agatston method, and patients were categorized as true severe or pseudo-severe according to sex-specific calcium thresholds. Of the patients, 57 were classified as true severe and 28 as pseudo-severe aortic stenosis. Results: Patients with higher calcification burden had significantly elevated serum uric acid levels (6.77 ± 1.57 vs. 5.08 ± 1.10 mg/dL, *p* < 0.001). Serum uric acid showed a modest correlation with aortic valve calcium score (ρ = 0.339, *p* = 0.002) and remained independently associated with CT-defined true severe low-flow, low-gradient aortic stenosis in multivariable analysis. ROC analysis yielded an area under the curve of 0.823 and identified a serum uric acid threshold of 5.45 mg/dL associated with a greater likelihood of CT-defined true severe low-flow, low-gradient aortic stenosis. Conclusions: Serum uric acid is associated with CT-derived aortic valve calcification and may provide insight into underlying fibro-calcific remodeling in this population.

## 1. Introduction

Low-flow, low-gradient (LF-LG) aortic stenosis (AS) with reduced left ventricular ejection fraction (LVEF) represents a complex and heterogeneous form of valvular heart disease [1,2]. Patients with classical LF–LG AS typically present with a small aortic valve area (AVA) (<1.0 cm^2^), low mean transvalvular gradient (<40 mmHg), reduced stroke volume index (<35 mL/m^2^), and impaired LVEF (<50%) [3,4]. In this setting, some patients have anatomically severe stenosis resulting from extensive fibro-calcific remodeling (true severe AS), whereas others exhibit functionally limited valve opening due to low transvalvular flow rather than fixed structural obstruction (pseudo-severe AS) [1,2,3,4]. These entities differ not only in their underlying pathophysiology but also in their therapeutic implications. Clinically, patients with true severe AS may benefit from valve intervention, whereas those with pseudo-severe disease are typically managed with therapies targeting ventricular dysfunction [3,4].

Dobutamine stress echocardiography (DSE) is commonly used to assess AS severity in low-flow conditions. However, its diagnostic accuracy depends on the presence of contractile reserve, which is absent in a substantial proportion of patients. As a result, DSE may yield inconclusive findings and persistent discordant grading [1,2,3,4]. Cardiac computed tomography (CT) derived aortic valve (AV) calcium scoring has therefore become an important complementary tool. Sex-specific Agatston thresholds reliably identify severe AS and correlate with disease progression and clinical outcomes [1,2,3,4]. Nevertheless, imaging-based approaches primarily capture the structural consequences of disease and may not fully reflect the biological processes driving valvular degeneration. A detailed understanding of the mechanisms underlying valve calcification is therefore essential for more accurate disease assessment and improved therapeutic strategies. Improved insight into these processes may refine the interpretation of disease severity and help explain the variability in clinical outcomes among patients with similar imaging findings. Such knowledge may also support the identification of novel therapeutic targets and facilitate a shift toward more personalized, mechanism-based management strategies beyond conventional imaging classification.

Oxidative stress plays a central role in AV calcification by promoting endothelial dysfunction, lipid oxidation, and osteogenic activation of valvular interstitial cells, leading to fibro-calcific remodeling and valve stiffening [5,6,7]. Uric acid, the final product of xanthine oxidase activity, reflects systemic oxidative stress and has been linked to endothelial dysfunction, lipoprotein oxidation, and tissue fibrosis [8,9,10]. Urate-related mechanisms may also contribute directly to valvular injury, as urate crystals and urate-driven inflammatory signaling are associated with tissue damage and progressive calcific changes within the AV [9,10]. In this context, serum uric acid (SUA) has been found to be associated with the severity of AS in previous studies [9,10]. However, this association has mainly been demonstrated using haemodynamic, flow-dependent grading rather than direct assessment of valvular calcification [9,10], raising uncertainty regarding its reflection of true structural valve disease. Accordingly, the relationship between SUA and the burden of valvular calcification, particularly in patients with LF–LG AS and reduced LVEF, remains incompletely understood. It remains unclear whether SUA reflects the degree of fibro-calcific remodeling or instead varies according to distinct pathophysiological phenotypes within this heterogeneous population. A better understanding of this association may provide additional biological context to imaging-based assessment and help refine disease characterization beyond conventional haemodynamic classification. In this regard, identifying biomarkers that reflect the underlying biological activity of valve disease may improve risk stratification, and may also offer insight into potential therapeutic targets aimed at modulating disease progression.

The aim of this study was to assess the association between SUA, as a marker of oxidative stress, and AV calcification in patients with LF–LG AS and reduced LVEF, to compare SUA levels between true severe and pseudo-severe disease, and to evaluate whether SUA reflects structural valve disease beyond flow-dependent haemodynamic severity.

## 2. Materials and Methods

### 2.1. Study Population

This retrospective observational study was conducted at a tertiary referral cardiology centre in accordance with the principles of the Declaration of Helsinki and was approved by the local institutional ethics committee (Decision No. 2026/05/1406, 10 March 2026). Consecutive patients evaluated between 2022 and 2025 with LF–LG AS and reduced LVEF were screened for inclusion.

All patients underwent comprehensive clinical evaluation, transthoracic echocardiography (TTE), and non-contrast cardiac CT for quantification of AV calcification using the Agatston method [11]. TTE examinations were performed by experienced cardiologists in accordance with current guideline recommendations. CT datasets were analysed offline by observers blinded to clinical and laboratory data, using standardized acquisition and analysis protocols.

Classical LF–LG AS was defined as AVA < 1.0 cm^2^, mean transvalvular gradient < 40 mmHg, stroke volume index < 35 mL/m^2^, and LVEF < 50% [4]. Patients were stratified into true severe and pseudo-severe LF–LG AS groups based on the AV calcium burden quantified by CT. Agatston calcium scores > 2000 AU in men and >1200 AU in women were considered indicative of true severe AS [4].

Demographic characteristics, comorbidities, medication use, and laboratory parameters were obtained from electronic medical records at the time of index evaluation. Blood samples were collected under fasting conditions, and SUA levels were measured using a standard automated enzymatic assay in the central laboratory. The neutrophil-to-lymphocyte ratio (NLR) was calculated by dividing the absolute neutrophil count by the absolute lymphocyte count. The systemic immune-inflammation index (SII) was calculated as platelet count × neutrophil count/lymphocyte count.

Patients with active inflammatory or infectious diseases, malignancy, haematological disorders, gout, severe hepatic dysfunction, end-stage renal disease, or current urate-lowering therapy were excluded. Patients with prior aortic valve intervention, congenital or rheumatic valve disease, or inadequate echocardiographic or CT image quality were also excluded.

### 2.2. Statistical Analysis

Continuous variables are presented as mean ± standard deviation and were compared between groups using independent-samples *t* tests or non-parametric tests when appropriate. Categorical variables are reported as counts and percentages and were compared using the chi-square test. Correlations between continuous variables were assessed using Spearman rank correlation analysis. Independent predictors of true severe LF–LG AS were identified using multivariable binary logistic regression analysis. The ability of SUA to identify CT-defined true severe LF-LG AS was evaluated using receiver-operating characteristic (ROC) curve analysis. A two-sided *p* value < 0.05 was considered statistically significant. All statistical analyses were performed using IBM SPSS Statistics version 26.0 (IBM Corp., Armonk, NY, USA).

## 3. Results

Eighty-five patients were included in the final analysis. Based on CT calcium thresholds, 28 patients were classified as having pseudo-severe LF–LG AS and 57 as true severe LF–LG AS.

Baseline clinical characteristics, laboratory findings, and inflammatory indices, including NLR and SII, were comparable between the two groups (Table 1). Patients with true severe LF–LG AS showed more advanced haemodynamic obstruction. AVA was smaller in the true severe group (0.70 ± 0.12 vs. 0.77 ± 0.10 cm^2^, *p* = 0.008), while both mean and peak transvalvular gradients were higher (mean gradient 26.3 ± 4.7 vs. 22.2 ± 6.0 mmHg, *p* = 0.001; peak gradient 44.6 ± 8.4 vs. 39.5 ± 10.2 mmHg, *p* = 0.016).

**Figure 1:**
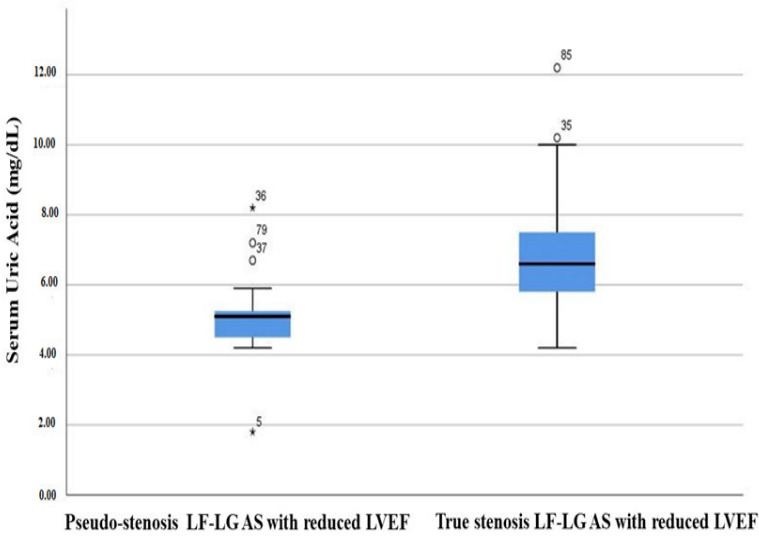
Distribution of serum uric acid levels according to aortic valve calcification burden in patients with low-flow, low-gradient aortic stenosis and reduced left ventricular ejection fraction. Group comparisons were performed using the independent samples *t*-test. Serum uric acid levels were higher in patients with greater calcification burden.

C-reactive protein (CRP) levels were higher in the true severe LF-LG AS group on unadjusted analysis (13.87 ± 15.36 vs. 6.61 ± 7.32 mg/L, *p* = 0.004). SUA levels were also significantly higher in patients with true severe LF–LG AS compared with those with pseudo-severe disease (6.77 ± 1.57 vs. 5.08 ± 1.10 mg/dL, *p* < 0.001) (Figure 1). In multivariable logistic regression analysis, serum uric acid remained independently associated with true severe LF–LG AS, whereas CRP was not independently associated with the outcome (Table 2).

**Figure 2:**
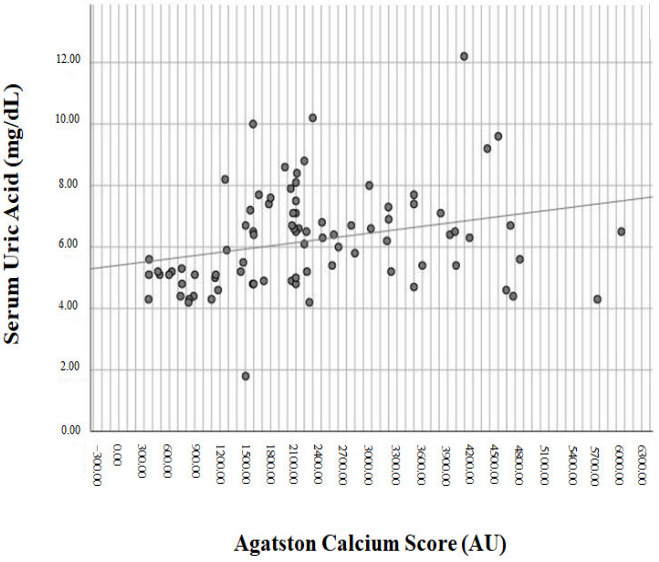
Relationship between serum uric acid levels and CT-derived Agatston aortic valve calcium score. Serum uric acid showed a modest positive correlation with calcification burden in patients with low-flow, low-gradient aortic stenosis.

Across the entire cohort, SUA demonstrated a weak to moderate positive correlation with AV calcium score (Spearman ρ = 0.339, *p* = 0.002), indicating an association between higher SUA levels and greater anatomical valvular calcification burden (Figure 2). No significant correlations were observed between SUA and AVA or transvalvular gradients, suggesting a closer relationship with structural calcification than with instantaneous haemodynamic severity (Table 3). B-type natriuretic peptide levels were numerically higher in the true severe group but did not reach statistical significance.

**Figure 3:**
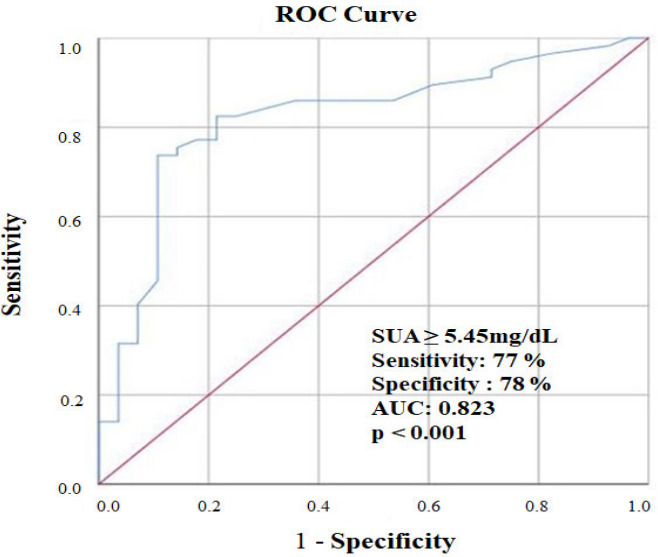
ROC curve illustrating the performance of serum uric acid levels in identifying higher aortic valve calcification burden in patients with low-flow, low-gradient aortic stenosis. AUC: Area under the curve; ROC: Receiver-operating characteristic; SUA: Serum uric acid.

ROC analysis yielded an area under the curve (AUC) of 0.823 and identified a SUA threshold of 5.45 mg/dL associated with a greater likelihood of true severe LF-LG AS. Internal validation using bootstrap resampling (5000 iterations) yielded a bootstrap-corrected AUC of 0.822 (95% CI: 0.718–0.910). A threshold of >5.45 mg/dL was associated with 77% sensitivity and 78% specificity for identifying true severe disease (Figure 3).

Overall, higher SUA levels were consistently associated with greater AV calcification burden across the cohort.

## 4. Discussion

Among patients with LF-LG AS and reduced LVEF, those with pronounced AV calcification exhibited higher SUA levels despite similar clinical characteristics and ventricular function. SUA was modestly correlated with AV calcium score, suggesting an association with underlying fibro-calcific valve remodeling rather than solely the haemodynamic consequences of low flow. In this context, SUA was independently associated with greater valvular calcification burden.

Oxidative stress plays a central role in AV calcification by promoting endothelial dysfunction, lipid oxidation, and osteogenic activation of valvular interstitial cells [5,6,7]. Early endothelial injury on the aortic side of the valve facilitates infiltration of lipids such as LDL and lipoprotein(a), which undergo oxidative modification and trigger local inflammatory and pro-calcific signalling [5,6,7]. Excess reactive oxygen species generated by xanthine oxidase and NADPH oxidases further amplify this process by reducing nitric oxide bioavailability and enhancing oxidative lipid modification [5,6,7,12]. These mechanisms promote osteogenic signalling within the valve, leading to progressive fibro-calcific remodelling and leaflet stiffening [5,6,7]. As calcification advances, increasing mechanical stress further amplifies oxidative injury, creating a self-perpetuating cycle of fibrosis and calcification [6,7].

Uric acid is the final product of xanthine oxidase activity and therefore reflects increased enzymatic activity and a systemic pro-oxidant state [8,9]. Accordingly, elevated SUA may serve as a surrogate marker of oxidative stress burden. Epidemiological studies have consistently associated elevated SUA levels with increased cardiovascular risk, including hypertension, coronary artery disease, heart failure, peripheral artery disease, chronic kidney disease, and cardiovascular mortality [8]. These associations are thought to involve SUA-related endothelial dysfunction, oxidative stress, and atherosclerotic processes, although causality has not been established [8]. Overall, SUA appears to be a stable and reproducible marker of cardiovascular disease burden across multiple clinical settings. SUA has also been reported to be associated with AS severity [9,10]; however, this relationship has mainly been demonstrated using haemodynamic classifications based on transvalvular pressure gradients rather than direct quantification of AV calcification [9,10]. This raises the possibility that the observed association is partly influenced by flow-dependent haemodynamic conditions rather than the underlying structural valve disease process. Previous studies have therefore been unable to fully distinguish whether the association between SUA and AS severity reflects haemodynamic consequences of advanced stenosis or the fibro-calcific remodeling process itself. To address this limitation, we evaluated SUA in relation to AV calcification using cardiac CT-derived calcium scoring, which provides a direct and objective measure of fibro-calcific remodelling [4,11,13]. Patients with classical LF-LG AS and reduced LVEF were stratified into true severe and pseudo-severe AS according to CT-derived aortic valve calcium burden. SUA was significantly associated with CT-derived aortic valve calcium burden and was higher in patients with true severe AS. Despite a broadly similar degree of left ventricular systolic dysfunction across the cohort and the potential influence of heart failure-related mechanisms on SUA levels, SUA remained significantly associated with calcium burden. These findings suggest that elevated SUA levels may reflect processes related to valvular fibro-calcific remodeling beyond the haemodynamic and metabolic consequences of heart failure.

In our study, conventional inflammatory markers, including leukocyte count, NLR, and SII, were not significantly associated with the severity of AV calcification. Although CRP levels were higher on unadjusted analysis, this association did not persist after multivariable adjustment. While previous studies have reported associations between inflammatory markers and haemodynamic measures of AS severity [14], our findings suggest that these markers may not adequately reflect the underlying fibro-calcific disease process in LF-LG AS. In contrast, SUA remained independently associated with greater calcific burden. This finding suggests that oxidative stress-related pathways may be more closely linked to valvular fibro-calcific remodeling than nonspecific systemic inflammatory markers. Moreover, experimental studies have suggested that urate-related mechanisms, including urate crystal deposition and urate-mediated inflammatory signalling, may directly contribute to valvular injury and the progression of AV calcification [10]. Therefore, our findings support an association between SUA and valvular calcification in LF-LG AS and raise the possibility that uric acid may be involved in the biological processes underlying calcific valve remodeling.

From a clinical perspective, ROC analysis identified a SUA threshold of 5.45 mg/dL associated with a greater likelihood of true severe LF–LG AS. In LF–LG AS, haemodynamic evaluation is particularly challenging due to low-flow conditions, which may lead to underestimation of transvalvular gradients and obscure the true severity of valvular obstruction. In this setting, SUA may provide complementary information that more closely reflects the underlying fibro-calcific valve biology, beyond load-dependent echocardiographic parameters. Importantly, while cardiac CT–derived AV calcium scoring remains the reference standard for the anatomical assessment of valvular calcification [1,2,3,4], SUA may offer additional value as a readily available biomarker reflecting systemic oxidative and metabolic stress, potentially capturing biological disease activity that is not directly assessed by imaging alone. Notably, the optimal SUA threshold identified in the present study (5.45 mg/dL) was lower than conventional definitions of hyperuricaemia, suggesting that the observed association with valvular calcification may extend beyond clinically overt hyperuricaemia. These findings may have implications for risk stratification in AS. SUA may help identify patients with more advanced or biologically active valvular remodelling, particularly in cases where echocardiographic measures are borderline or discordant. As such, it may serve as an adjunct to imaging, supporting a more integrated approach to disease assessment.

From a broader perspective, the observed association between SUA and valvular calcification may provide additional insight into the biological mechanisms underlying calcific AV disease. Our findings should not be interpreted as supporting a diagnostic role for SUA or as a substitute for CT-derived calcium scoring, which remains the reference method for assessing valvular calcification in LF-LG AS. Rather, SUA may reflect oxidative stress-related processes involved in fibro-calcific valve remodeling. In this context, the present findings support the possibility that the association between SUA and aortic stenosis extends beyond haemodynamic severity alone and may be linked to the underlying structural disease process. These observations may generate hypotheses regarding the role of metabolic and oxidative pathways in disease progression and could provide a rationale for future investigations exploring whether modulation of uric acid metabolism or related enzymatic pathways, such as xanthine oxidase activity, influences the progression of valvular calcification. However, these interpretations remain hypothesis-generating, and prospective mechanistic studies are needed to clarify causality and potential therapeutic relevance.

This study has several limitations. The retrospective, single-center design and relatively small sample size limit causal inference and generalisability. The cross-sectional nature of the analysis precludes assessment of temporal relationships between SUA levels and progression of valvular calcification. In addition, SUA levels may be influenced by renal function, diet, and medications; although renal function was comparable between groups, residual confounding cannot be fully excluded. Moreover, disease classification was based on CT-derived calcium thresholds, which inherently reflect differences in calcification burden. Nevertheless, the continuous association observed between SUA and AV calcium score across the entire cohort supports a true underlying biological relationship rather than a purely categorical effect. No formal correction for multiple comparisons was applied to the correlation analyses; therefore, these findings should be considered exploratory and interpreted with appropriate caution. Although internal validation using bootstrap resampling demonstrated minimal optimism in the estimated AUC, the identified SUA threshold and diagnostic performance have not been externally validated and should therefore be interpreted with appropriate caution. Finally, these findings should be validated in prospective, larger-scale studies.

## 5. Conclusions

This study demonstrates that elevated SUA is associated with greater AV calcification burden in patients with LF–LG AS and reduced LVEF. SUA may provide insight into the biological processes underlying fibro-calcific remodeling of the AV. These findings highlight the potential relevance of metabolic and oxidative pathways in calcific AV disease. Further prospective studies are warranted to clarify the role of SUA in disease progression and clinical risk stratification.

## Figures and Tables

**Table 1:** Baseline Clinical, Echocardiographic, and Laboratory Characteristics.

Variable (Unit)	Pseudo-Severe LF-LG AS (*n* = 28)	True-Severe LF-LG AS (*n* = 57)	*p* Value
Age (years)	80.43 ± 5.34	78.60 ± 6.11	0.180
Male sex, *n* (%)	17 (60.7)	37 (64.9)	0.955
Hypertension, *n* (%)	23 (82.1)	52 (91.2)	0.222
Diabetes mellitus, *n* (%)	9 (32.1)	27 (47.4)	0.182
Coronary artery disease, *n* (%)	19 (67.9)	45 (78.9)	0.265
Diuretic use, *n* (%)	15 (53.6)	37 (64.9)	0.313
LVEF (%)	39.64 ± 10.09	37.02 ± 7.61	0.230
Aortic valve area (cm^2^)	0.77 ± 0.10	0.70 ± 0.12	0.008
Mean gradient (mmHg)	22.18 ± 5.99	26.33 ± 4.66	0.001
Peak gradient (mmHg)	39.50 ± 10.20	44.60 ± 8.35	0.016
Agatston calcium score (AU)	1015.25 ± 433.35	2923.63 ± 1082.38	<0.001
White blood cells (×10^9^/L)	7.53 ± 1.69	7.17 ± 2.31	0.462
Neutrophils (×10^9^/L)	5.08 ± 1.48	4.96 ± 1.83	0.759
Lymphocytes (×10^9^/L)	1.73 ± 0.61	1.47 ± 0.64	0.071
Platelets (×10^9^/L)	234.11 ± 74.63	233.67 ± 74.31	0.980
Hemoglobin (g/dL)	11.92 ± 1.49	11.24 ± 2.13	0.095
Neutrophil-to-lymphocyte ratio	3.29 ± 1.48	4.18 ± 2.97	0.135
Systemic immune-inflammation index	758.24 ± 348.76	975.19 ± 754.59	0.152
C-reactive protein (mg/L)	6.61 ± 7.32	13.87 ± 15.36	0.004
Blood urea nitrogen (mg/dL)	24.82 ± 10.78	27.12 ± 10.46	0.348
Creatinine (mg/dL)	1.12 ± 0.59	1.31 ± 1.02	0.369
Estimated GFR (mL/min/1.73 m^2^)	63.32 ± 18.29	59.60 ± 19.62	0.403
AST (U/L)	22.82 ± 10.46	25.95 ± 17.39	0.384
ALT (U/L)	18.46 ± 12.98	21.42 ± 27.17	0.587
Serum uric acid (mg/dL)	5.08 ± 1.10	6.77 ± 1.57	<0.001
BNP (pg/mL)	744.00 ± 992.50	1254.39 ± 1276.11	0.067

ALT: Alanine Aminotransferase AST: Aspartate Aminotransferase BNP: B-type Natriuretic Peptide LF-LG AS: Low Flow-Low Gradient Aortic Stenosis LVEF: Left Ventricular Ejection Fraction.

**Table 2:** Multivariable Analysis of Factors Associated with CT-Defined True Severe LF-LG AS.

Variable	B	Standard Error	Wald	*p* Value	Odds Ratio
SUA	1.091	0.299	13.295	≤0.001	2.977
CRP	0.051	0.034	2.230	0.135	1.052
Constant	−6.069	1.682	13.013	≤0.001	0.002

CRP: C-reactive protein, SUA: Serum Uric Acid.

**Table 3:** Correlation of SUA with echocardiographic parameters and AV calcium score.

	Correlation Coefficient (ρ)	*p*-Value
Aortic Valve Area	−0.102	NS
Peak AV Gradient	0.096	NS
Mean AV Gradient	0.167	NS
AV calcium score (Agatston)	0.339	0.002

AV: Aortic valve; NS: Not significant; SUA: Serum Uric Acid.

## Data Availability

The data supporting the findings of this study are available from the corresponding author upon reasonable request. The data are not publicly available due to privacy and ethical restrictions.

## Abbreviations

The following abbreviations are used in this manuscript:

AS	Aortic stenosis
AUC	Area under the curve
AV	Aortic valve
AVA	Aortic valve area
CRP	C-reactive protein
CT	Computed tomography
DSE	Dobutamine stress echocardiography
LF-LG	Low-flow, low-gradient
LVEF	Left ventricular ejection fraction
NLR	Neutrophil-to-lymphocyte ratio
ROC	Receiver operating characteristic
SUA	Serum uric acid
SII	Systemic immune-inflammation index
